# Supplementary material for: RNF185 regulates proteostasis in Ebolavirus infection by crosstalk between the calnexin cycle, ERAD, and reticulophagy
Source: Nat Commun. 2022 Oct 12;13:6007. doi: 10.1038/s41467-022-33805-9 (PMC9554868; doi:10.1038/s41467-022-33805-9)
Supplement: Supplementary file 2 — Reporting Summary [file 41467_2022_33805_MOESM2_ESM.pdf]

nature portfolio

Corresponding author(s): Zheng, Yong-Hui

Last updated by author(s): 9/15/2022

Reporting Summary

Nature Portfolio wishes to improve the reproducibility of the work that we publish. This form provides structure for consistency and transparency in reporting. For further information on Nature Portfolio policies, see our Editorial Policies and the Editorial Policy Checklist.

Statistics

For all statistical analyses, confirm that the following items are present in the figure legend, table legend, main text, or Methods section.

n/a	Confirmed	
☐	☒	The exact sample size (*n*) for each experimental group/condition, given as a discrete number and unit of measurement
☐	☒	A statement on whether measurements were taken from distinct samples or whether the same sample was measured repeatedly
☐	☒	The statistical test(s) used AND whether they are one- or two-sided<br>*Only common tests should be described solely by name; describe more complex techniques in the Methods section.*
☒	☐	A description of all covariates tested
☒	☐	A description of any assumptions or corrections, such as tests of normality and adjustment for multiple comparisons
☐	☒	A full description of the statistical parameters including central tendency (e.g. means) or other basic estimates (e.g. regression coefficient) AND variation (e.g. standard deviation) or associated estimates of uncertainty (e.g. confidence intervals)
☐	☒	For null hypothesis testing, the test statistic (e.g. *F*, *t*, *r*) with confidence intervals, effect sizes, degrees of freedom and *P* value noted<br>*Give P values as exact values whenever suitable.*
☒	☐	For Bayesian analysis, information on the choice of priors and Markov chain Monte Carlo settings
☒	☐	For hierarchical and complex designs, identification of the appropriate level for tests and full reporting of outcomes
☒	☐	Estimates of effect sizes (e.g. Cohen's *d*, Pearson's *r*), indicating how they were calculated

Our web collection on statistics for biologists contains articles on many of the points above.

Software and code

Policy information about availability of computer code

Data collection	No software was used for data collection.
Data analysis	GraphPad Prism (Version 9.3.1) was used for the data analysis. Adobe Photoshop 2021 was used to analyze western blot images. Adobe Illustrator 2021 was used to create figures. SnapGene 6.0.2 was used to create vector maps. BioRender was used for making the model (https://biorender.com). qPCR results were analyzed using Bio-Rad CFX Manager 3.1. Western blots were quantified with ImageJ Launcher 1.4.3.67 (https://imagej.nih.gov/ij/). Confocal images were analyzed using ZEISS Zen Software version 2.1.

For manuscripts utilizing custom algorithms or software that are central to the research but not yet described in published literature, software must be made available to editors and reviewers. We strongly encourage code deposition in a community repository (e.g. GitHub). See the Nature Portfolio guidelines for submitting code & software for further information.

Data

Policy information about availability of data

All manuscripts must include a data availability statement. This statement should provide the following information, where applicable:

- Accession codes, unique identifiers, or web links for publicly available datasets
- A description of any restrictions on data availability
- For clinical datasets or third party data, please ensure that the statement adheres to our policy

A source data file will be provided with this paper.

Field-specific reporting

Please select the one below that is the best fit for your research. If you are not sure, read the appropriate sections before making your selection.

☒ Life sciences ☐ Behavioural & social sciences ☐ Ecological, evolutionary & environmental sciences

For a reference copy of the document with all sections, see nature.com/documents/nr-reporting-summary-flat.pdf

Life sciences study design

All studies must disclose on these points even when the disclosure is negative.

Sample size	At each point of data collection (Fig.2A, Fig.2B, Fig.2C, Fig.4H), at least three samples were collected for data analysis. No significant variation was observed, indicating the sample size was sufficient.
Data exclusions	No data were excluded.
Replication	Experiments were repeated three times. All attempts at replication were successful.
Randomization	As this was a hypothesis-driven study without intervention, no randomization was necessary.
Blinding	As this was a hypothesis-driven study without intervention, blinding design was not necessary.

Reporting for specific materials, systems and methods

We require information from authors about some types of materials, experimental systems and methods used in many studies. Here, indicate whether each material, system or method listed is relevant to your study. If you are not sure if a list item applies to your research, read the appropriate section before selecting a response.

Materials & experimental systems

n/a	Involved in the study	
☐	☒	Antibodies
☐	☒	Eukaryotic cell lines
☒	☐	Palaeontology and archaeology
☒	☐	Animals and other organisms
☒	☐	Human research participants
☒	☐	Clinical data
☒	☐	Dual use research of concern

Methods

n/a	Involved in the study	
☒	☐	ChIP-seq
☐	☒	Flow cytometry
☒	☐	MRI-based neuroimaging

Antibodies

Antibodies used

EBOV-GP Antibody, Rabbit PAb, Antigen Affinity Purified Sino Biological 400442-T48
EBOV-VP40 Antibody, Rabbit PAb, Antigen Affinity Purified Antibody Sino Biological 400446-T48
Mouse monoclonal anti-HIV-1 gp120 NIH AIDS Reagent Program 521
Mouse monoclonal anti-VSV G SIGMA SAB4200695
Anti-H5HA Sino Biological 11048-RP02
Anti-FLAG M2 SIGMA F3165-5MG
Anti-HA SIGMA H3663
Anti-Myc-Tag (9B11) Mouse mAb Cell Signaling 2276
Anti-His-HRP Proteintech 66005
Anti-ERp57 (G117) Antibody Cell Signaling 2881S
Anti-CALR Antibody ENZO ADI-SPA-865-F
Anti-CANX Antibody ENZO ADI-SPA-600-F
Anti-RNF26 Antibody Sino Biological 203155-T32
Anti-GFP-Tag Proteintech 50430-2-AP
Anti-Lamp2A Abcam ab18528
Anti-LC3-I SIGMA L7543
Anti-P62 SIGMA P0067
Anti-HDAC6 Sino Biological 100768-T08
Anti-beta-Actin SIGMA A5441-5ML
HRP-goat anti-mouse Jackson JAC-115-035-003
HRP-goat anti-rabbit Jackson JAC-111-035-003
Alexa Fluor 647-conjugated donkey anti-rabbit IgG Invitrogen A31573
Alexa Fluor 647-conjugated donkey anti-mouse IgG Invitrogen A31571

HIV-1 p24Gag ELISA kit NOVUS NBP2-79359
HiBiT-blotting kit Promega N2410

Validation

Validation was based on data sheets provided by the manufactures, and further confirmed by our experiments.

Eukaryotic cell lines

Policy information about cell lines

Cell line source(s)

HEK293T ATCC CRL-3216
Hela ATCC CRM-CCL-2
Vero E6 ATCC CRL-1586
A549 ATCC CRM-CCL-185
HepG2 ATCC HB-8065
THP-1 ATCC TIB-202
HEK293T-CANK-KO (Wang et al. JBC, 2017)
HEK293T-CALR-KO (Wang et al. JBC, 2017)
HEK293T-CANX/CALR-KO (Wang et al. JBC, 2017)
HEK293T-PDIA3-KO (Wang et al. Autophagy, 2022)
HEK293T-ATG3-KO (Wang et al. Autophagy, 2022)
HEK293T-ATG5-KO (Wang et al. Autophagy, 2022)
HEK293T-SQSTM1-KO (Wang et al. Autophagy, 2022)
HeLa-SQSTM1-KO (Wang et al. Autophagy, 2022)
A549-SQSTM1-KO (Wang et al. Autophagy, 2022)
HeLa-HDAC6-KO (Wang et al. Autophagy, 2022)
HEK293T-RNF26-KO
HEK293T-PDIA3/CALR-KO
HEK293T-PDIA3/CANX-KO

Authentication

HEK293T-RNF26-KO, HEK293T-PDIA3/CALR-KO, and HEK293T-PDIA3/CANX-KO were created by CRISPR/Cas9 and confirmed by genome sequencing and western blotting. All the other cell lines were from ATCC and our previously works that have clear track-records, so they were not recently authenticated.

Mycoplasma contamination

All these cell lines were negative for mycoplasma contamination.

Commonly misidentified lines
(See ICLAC register)

No commonly misidentified cell lines were used.

Flow Cytometry

Plots

Confirm that:

- [ ] The axis labels state the marker and fluorochrome used (e.g. CD4-FITC).
- [ ] The axis scales are clearly visible. Include numbers along axes only for bottom left plot of group (a 'group' is an analysis of identical markers).
- [ ] All plots are contour plots with outliers or pseudocolor plots.
- [ ] A numerical value for number of cells or percentage (with statistics) is provided.

Methodology

Sample preparation

No flow data in this paper.

Instrument

Identify the instrument used for data collection, specifying make and model number.

Software

Describe the software used to collect and analyze the flow cytometry data. For custom code that has been deposited into a community repository, provide accession details.

Cell population abundance

Describe the abundance of the relevant cell populations within post-sort fractions, providing details on the purity of the samples and how it was determined.

Gating strategy

Describe the gating strategy used for all relevant experiments, specifying the preliminary FSC/SSC gates of the starting cell population, indicating where boundaries between "positive" and "negative" staining cell populations are defined.

- [ ] Tick this box to confirm that a figure exemplifying the gating strategy is provided in the Supplementary Information.
